# Reactive Oxygen Species-Responsive Signaling Networks and Oxidative Stress Adaptation in Critical Priority Fungal Pathogens

**DOI:** 10.3390/jof12080620

**Published:** 2026-08-19

**Authors:** Raichal B. George, Hari Govind Pradeep, Nandaja Adikaledath Mana, Nandana Raj, Pavithra Praveen, Rithik P. Harish, Nimisha Mahesh, Dhannya Renuka, Bipin G. Nair, Geetha B. Kumar, Jayalekshmi Haripriyan

**Affiliations:** Amrita School of Biotechnology, Amrita Vishwa Vidhyapeetham, Clappana P.O., Kollam 690525, Kerala, India

**Keywords:** reactive oxygen species, oxidative stress, *Cryptococcus neoformans*, *Candida auris*, *Aspergillus fumigatus*, *Candida albicans*, signaling pathways

## Abstract

Invasive fungal diseases (IFDs) are a global health threat, especially among immunocompromised populations, due to their high mortality rates and the increasing prevalence of antifungal resistance. In recognition of this threat, the World Health Organization (WHO) has designated *Cryptococcus neoformans*, *Candida auris*, *Aspergillus fumigatus*, and *Candida albicans* as critical-priority fungal pathogens. During host infection, host-derived reactive oxygen species (ROS) function as potent antimicrobial molecules, whereas fungal-derived ROS act as intracellular signaling mediators regulating oxidative stress adaptation, metabolism, virulence, and antifungal tolerance. Although oxidative stress responses have been extensively investigated in individual fungal pathogens, a comprehensive comparative analysis of oxidative stress signaling across these critical fungal pathogens remains limited. This review systematically compares oxidative stress sensing and signaling networks in the four WHO critical-priority fungal pathogens and classifies oxidative stress-associated pathways into conserved, and species-specific regulatory mechanisms. Conserved pathways, including HOG-MAPK, calcineurin, cAMP-PKA, cell wall integrity, and thioredoxin-dependent signaling, are discussed alongside pathogen-specific adaptations that promote biofilm formation, capsule and melanin production, polarized growth, morphogenesis, immune evasion, and antifungal resistance. By integrating conserved and divergent oxidative stress signaling mechanisms, this review provides a comparative framework that advances our understanding of fungal pathogenesis and highlights potential targets for the development of broad-spectrum and species-specific antifungal therapies.

## 1. Introduction

Infectious diseases remain a leading cause of mortality and disability worldwide [1]. Alongside this major global health threat, IFDs are rising, particularly among immunocompromised populations, causing approximately 3.8 million deaths annually [2]. The diagnosis and treatment of IFDs are challenged by limited access to quality diagnostics and inadequate treatment options as well as the emergence of antifungal resistance in many healthcare settings [3]. In response to these concerns, WHO released its first Fungal Priority Pathogen List (FPPL) in 2022, which classifies 19 fungal pathogens into three priority groups: critical, high, and medium [4]. Among these, the critical group included *Cryptococcus neoformans*, *Candida auris*, *Aspergillus fumigatus*, and *Candida albicans* [5]. Although these four pathogens differ markedly in morphology, ecological niches, life cycles, and mechanisms of pathogenesis, they are all exposed to host-derived oxidative stresses during infection and rely on conserved yet functionally diversified ROS-responsive signaling pathways for survival [6]. *Cryptococcus neoformans* is an encapsulated opportunistic yeast that primarily causes cryptococcal meningitis and pulmonary cryptococcosis in immunocompromised individuals. Its virulence largely depends on polysaccharide capsule formation, melanin biosynthesis, and the ability to survive within host macrophages, all of which contribute to resistance against oxidative stress [7]. *Candida auris* has rapidly emerged as a multidrug-resistant nosocomial pathogen responsible for invasive bloodstream and healthcare-associated infections with high mortality rates. Its remarkable persistence on environmental surfaces, resistance to multiple classes of antifungal agents, and efficient transmission within healthcare settings have made it a global public health concern [8]. *Aspergillus fumigatus*, the predominant cause of invasive aspergillosis, possesses specialized oxidative stress adaptation mechanisms that facilitate filamentous growth, tissue invasion, and survival within the hostile host environment [9]. *Candida albicans*, although a common human commensal organism, remains the leading opportunistic fungal pathogen responsible for both mucosal and systemic candidiasis through its ability to undergo morphogenetic switching, form persistent biofilms, and evade host immune defenses [10]. Collectively, these four fungal pathogens represent diverse models of fungal biology and pathogenicity while sharing common challenges associated with oxidative stress adaptation during host infection [11,12,13].

During infection, activated macrophages and neutrophils generate high levels of ROS through the oxidative burst as an essential component of innate immune defense aimed at eliminating invading fungal pathogens [14]. To counteract these host-derived oxidants, pathogenic fungi have evolved sophisticated oxidative stress-responsive signaling pathways that detect alterations in intracellular redox homeostasis and activate antioxidant defense systems, including superoxide dismutases, catalases, glutathione, thioredoxin, and peroxiredoxin-dependent pathways [15]. Beyond their antimicrobial activity, intracellular ROS also function as signaling molecules that regulate fungal metabolism, morphogenesis, stress adaptation, biofilm formation, mitochondrial homeostasis, and virulence [16]. Consequently, oxidative stress signaling represents a fundamental determinant of fungal survival, pathogenicity, immune evasion, and antifungal tolerance [17].

The biological effects of ROS are highly concentration dependent. At low to moderate concentrations, ROS function as intracellular second messengers that regulate growth, metabolism, morphogenesis, and adaptive stress responses [18]. However, excessive ROS accumulation overwhelms antioxidant defense mechanisms, resulting in oxidative damage to proteins, lipids, and nucleic acids that ultimately compromises cellular viability [19] Importantly, no universal quantitative threshold distinguishes physiological ROS signaling from oxidative toxicity because ROS responses vary according to fungal species, intracellular compartmentalization, developmental stage, metabolic state, and environmental conditions [14]. Consequently, the transition from ROS-mediated signaling to oxidative stress is dynamic and context dependent [20].

Although oxidative stress responses have been extensively investigated in individual fungal pathogens, a comprehensive comparative understanding of oxidative stress-responsive signaling networks across the four WHO critical-priority fungal pathogens remains limited [11,21,22,23,24,25,26]. Most available studies focus on species-specific mechanisms, whereas comparatively little attention has been given to the evolutionary conservation, functional diversification, and integration of signaling pathways governing oxidative stress adaptation, fungal virulence, immune evasion, and antifungal resistance [18]. Furthermore, the relative contributions of conserved and pathogen-specific signaling networks to fungal pathogenesis remain incompletely understood, limiting the identification of broadly effective antifungal targets.

Unlike previous reviews that primarily discuss oxidative stress responses in individual fungal species, this review adopts a comparative framework encompassing *Cryptococcus neoformans*, *Candida auris*, *Aspergillus fumigatus*, and *Candida albicans*. We systematically classify oxidative stress-responsive signaling pathways according to their mechanisms of activation and evolutionary conservation, distinguishing direct ROS-sensing pathways from indirect oxidative stress-responsive signaling networks, and further categorizing these pathways as conserved, or species-specific regulatory mechanisms. By integrating similarities and differences in oxidative stress signaling across these clinically important fungal pathogens, this review provides a comprehensive overview of the molecular networks governing fungal adaptation, virulence, immune evasion, and antifungal tolerance while highlighting potential targets for the development of both broad-spectrum and pathogen-specific antifungal therapies.

## 2. Classification of Oxidative Stress Signaling Pathways

### 2.1. Direct and Indirect Oxidative Stress-Responsive Signaling Pathways

Oxidative stress adaptation in fungal pathogens is mediated through both direct ROS-sensing mechanisms and indirect stress-responsive signaling pathways [27]. Direct ROS-sensing pathways detect alterations in intracellular redox status through oxidation-sensitive proteins, thereby rapidly activating antioxidant defense responses. In contrast, indirect pathways are activated in response to cellular perturbations caused by oxidative stress, including protein misfolding, calcium imbalance, mitochondrial dysfunction, and cell wall damage [28]. Although these pathways do not directly sense ROS, they coordinate broader adaptive responses that enhance cellular survival and maintain homeostasis under oxidative stress. For conceptual clarity, the major oxidative stress-responsive signaling pathways discussed in this review are classified according to their primary modes of activation and physiological functions in Table 1.

### 2.2. Conserved and Species-Specific Oxidative Stress Signaling Pathways

Comparative analyses of oxidative stress-responsive signaling networks reveal both conserved stress adaptation mechanisms and species-specific regulatory features among *Cryptococcus neoformans*, *Candida auris*, *Aspergillus fumigatus*, and *Candida albicans*. These similarities and differences reflect the evolutionary adaptation of each pathogen to distinct ecological niches and host environments [29,30,31,32]. Conserved signaling pathways include the HOG-MAPK, calcineurin, cAMP-PKA, and thioredoxin-dependent systems, which together constitute the core oxidative stress response network in pathogenic fungi [14]. In *Cryptococcus neoformans*, the HOG-MAPK pathway functions in concert with the two-component signaling (TCS) system to regulate oxidative and osmotic stress responses, capsule formation, and virulence [32,33]. Calcineurin contributes to calcium homeostasis and thermotolerance, whereas the cAMP-PKA pathway links oxidative stress adaptation to metabolism, cellular growth, and capsule and melanin biosynthesis. The thioredoxin system further preserves intracellular redox homeostasis and protects cells against oxidative damage [34]. Several additional signaling pathways are conserved across fungal pathogens but have undergone functional diversification, including the Cap1/Yap1-Gpx3-Ybp1 regulatory module, Skn7, the cell wall integrity (CWI)-MAPK pathway, the unfolded protein response (UPR), and the TCS [35,36]. Although these pathways are present in multiple fungal species, they differ in their regulatory interactions, downstream target genes, and biological functions [35].

For example, Cap1-mediated oxidative stress signaling in *Candida albicans* is closely associated with morphogenetic switching and survival within macrophages, whereas related regulatory networks in *Candida auris* primarily promote oxidative stress tolerance, persistence, and adaptation to antifungal stress. Similarly, the CWI-MAPK pathway links oxidative stress to cell wall remodeling in several fungal pathogens, although its relative contribution to stress adaptation and virulence varies among species [12,36]. In contrast, some oxidative stress-associated signaling pathways exhibit clear species-specific adaptations. In *Aspergillus fumigatus*, the RacA-Nox pathway regulates localized ROS production required for polarized hyphal growth, conidiation, and fungal development [37]. These specialized mechanisms reflect the unique biology of filamentous fungi and are absent, or considerably less prominent in *Candida* species [38]. Overall, pathogenic fungi share a conserved core of oxidative stress-responsive signaling pathways while employing distinct regulatory strategies that support their diverse pathogenic lifestyles [14]. Understanding both the conserved and species-specific features of these signaling networks may facilitate the development of broad-spectrum as well as pathogen-specific antifungal therapies [39].

The conserved and species-specific oxidative stress-responsive signaling pathways discussed in this review are comparatively summarized in Table 2.

### 2.3. Cryptococcus neoformans

#### 2.3.1. ROS Signaling Pathways

*Cryptococcus neoformans* possesses a complex network of conserved signaling pathways that regulate virulence, morphogenesis, stress adaptation, and host–pathogen interactions [54]. These pathways form an integrated regulatory network that enables the fungus to rapidly adapt to host-associated stresses and establish infection [32]. Consequently, conserved fungal signaling modules represent promising targets for the development of broad-spectrum antifungal therapies [55].

#### 2.3.2. HOG-MAPK Signaling Pathway

The high osmolarity glycerol (HOG)-MAPK pathway is the principal signaling cascade governing oxidative stress adaptation in *Cryptococcus neoformans* [56]. Although initially characterized as a regulator of osmotic stress responses, it also plays a central role in coordinating cellular adaptation to ROS, ultraviolet irradiation, heavy metals, antifungal drugs, and other environmental stresses encountered during host infection [57]. Oxidative stress is sensed through a fungal-specific two-component signaling system comprising hybrid histidine kinases (Tco proteins), the phosphotransfer protein Ypd1, and the response regulator Ssk1 [58]. Upon exposure to hydrogen peroxide (H_2_O_2_) or other oxidative stressors, stress signals are transmitted from Ssk1 to the MAPKKK Ssk2, followed by sequential activation of the MAPK kinase Pbs2 and the MAPK Hog1. Following phosphorylation, Hog1 translocates to the nucleus, where it regulates the expression of genes involved in antioxidant defense, osmoadaptation, ergosterol biosynthesis, sulfur metabolism, and cellular detoxification [33]. Several antioxidant proteins function downstream of Hog1, including sulfiredoxin (Srx1), thioredoxins (Trx1 and Trx2), and peroxiredoxins (Tsa1), which collectively detoxify hydrogen peroxide and restore intracellular redox homeostasis [59]. Beyond oxidative stress adaptation, the HOG-MAPK pathway contributes to capsule biosynthesis, adaptation to osmotic and thermal stress, antifungal drug susceptibility, and fungal virulence [56]. Notably, unlike many other fungal pathogens, Hog1 in *Cryptococcus neoformans* is constitutively phosphorylated under non-stress conditions and undergoes transient dephosphorylation followed by rephosphorylation during oxidative stress, reflecting a distinctive mode of pathway regulation [28]. Recent studies have further expanded the role of ROS beyond stress adaptation by demonstrating its involvement in titan cell development, a key virulence trait of *Cryptococcus neoformans* [60]. The accumulation of endogenous ROS is required for titan-like cell formation, indicating that controlled ROS production functions as a signaling cue rather than merely a damaging by-product of metabolism [61]. Pharmacological inhibition of intracellular ROS prevented titan cell formation, suggesting that ROS-dependent signaling contributes to morphological differentiation and fungal adaptation during host infection [62]. These findings further reinforce the concept that oxidative stress-responsive signaling regulates multiple virulence-associated developmental processes in *C. neoformans*. Overall, the HOG-MAPK pathway integrates oxidative stress adaptation with capsule formation, thermotolerance, and host survival, underscoring its central role in the virulence of *Cryptococcus neoformans* [57].

#### 2.3.3. PKC-Mpk1 Cell Wall Integrity (CWI) Pathway

The protein kinase C (PKC)-mediated cell wall integrity (CWI) pathway enables *Cryptococcus neoformans* to maintain cell wall architecture during oxidative stress and other host-imposed environmental challenges. Oxidative damage compromises cell wall polysaccharides and membrane integrity, necessitating rapid repair to preserve cellular viability [63]. Activation of this pathway is initiated by phospholipase C-mediated lipid signaling, which generates diacylglycerol (DAG) and activates Pkc1 [64]. Pkc1 subsequently initiates a MAPK cascade comprising the MAPKKK Bck1, the MAPKK Mkk2, and the MAPK Mpk1 [65]. Following activation, Mpk1 regulates the expression of genes involved in β-1,3-glucan synthesis, chitin remodeling, membrane stability, and cell wall repair. Beyond maintaining cell wall integrity, the PKC-Mpk1 pathway contributes to thermotolerance, capsule attachment, melanin production, and resistance to oxidative and nitrosative stress. Mutants lacking *PKC1* exhibit severe defects in capsule formation, increased sensitivity to oxidative stress, impaired growth at 37 °C, and markedly attenuated virulence in animal infection models [66]. Functional crosstalk between the PKC-Mpk1 and calcineurin signaling pathways further enhances fungal survival under host-imposed stress conditions [67].

#### 2.3.4. Calcineurin Signaling Pathway

Calcineurin is a conserved Ca^2+^/calmodulin-dependent serine/threonine phosphatase that functions as a central regulator of stress adaptation in *Cryptococcus neoformans* [68,69]. Increased intracellular Ca^2+^ binds to calmodulin, resulting in calcineurin activation. Activated calcineurin dephosphorylates the transcription factor Crz1, promoting its nuclear translocation and the subsequent expression of genes involved in calcium homeostasis, cell wall maintenance, ion transport, membrane integrity, and stress adaptation [70]. Calcineurin is essential for fungal growth at mammalian body temperature (37 °C), intracellular survival within macrophages, and dissemination during cryptococcal infection [71]. Deletion of calcineurin components results in hypersensitivity to oxidative stress, impaired cell wall integrity, reduced thermotolerance, and complete loss of virulence [72]. Recent studies have expanded the functional roles of calcineurin beyond Crz1-mediated transcriptional regulation, which demonstrates that calcineurin also regulates stress granule dynamics and glycerol biosynthesis during cationic stress, indicating both transcriptional and post-translational mechanisms of stress adaptation [73]. These findings suggest that calcineurin coordinates multiple stress-responsive processes, including translational regulation, osmoadaptation, and cellular homeostasis, thereby enhancing the ability of *C. neoformans* to survive hostile host environments [74]. Furthermore, calcineurin inhibitors, such as FK506 (tacrolimus) and cyclosporine A, markedly enhance antifungal susceptibility, highlighting this pathway as an attractive therapeutic target for cryptococcal infections [75].

#### 2.3.5. Ras-cAMP-PKA Signaling Pathway

The Ras-cAMP (cAMP)-protein kinase A (PKA) signaling pathway coordinates fungal growth, metabolism, morphogenesis, and the expression of major virulence determinants in *Cryptococcus neoformans* [76]. Although this pathway does not directly sense oxidative stress, its activity is substantially influenced by oxidative stress through changes in cellular metabolism and environmental signaling [77]. Overall, the Ras-cAMP-PKA pathway integrates environmental sensing with oxidative stress adaptation and virulence-associated processes, thereby facilitating host adaptation and pathogenicity in *Cryptococcus neoformans* [54].

#### 2.3.6. Integration of Oxidative Stress Responsive Signaling Networks in *Cryptococcus neoformans*

**Figure 1:**
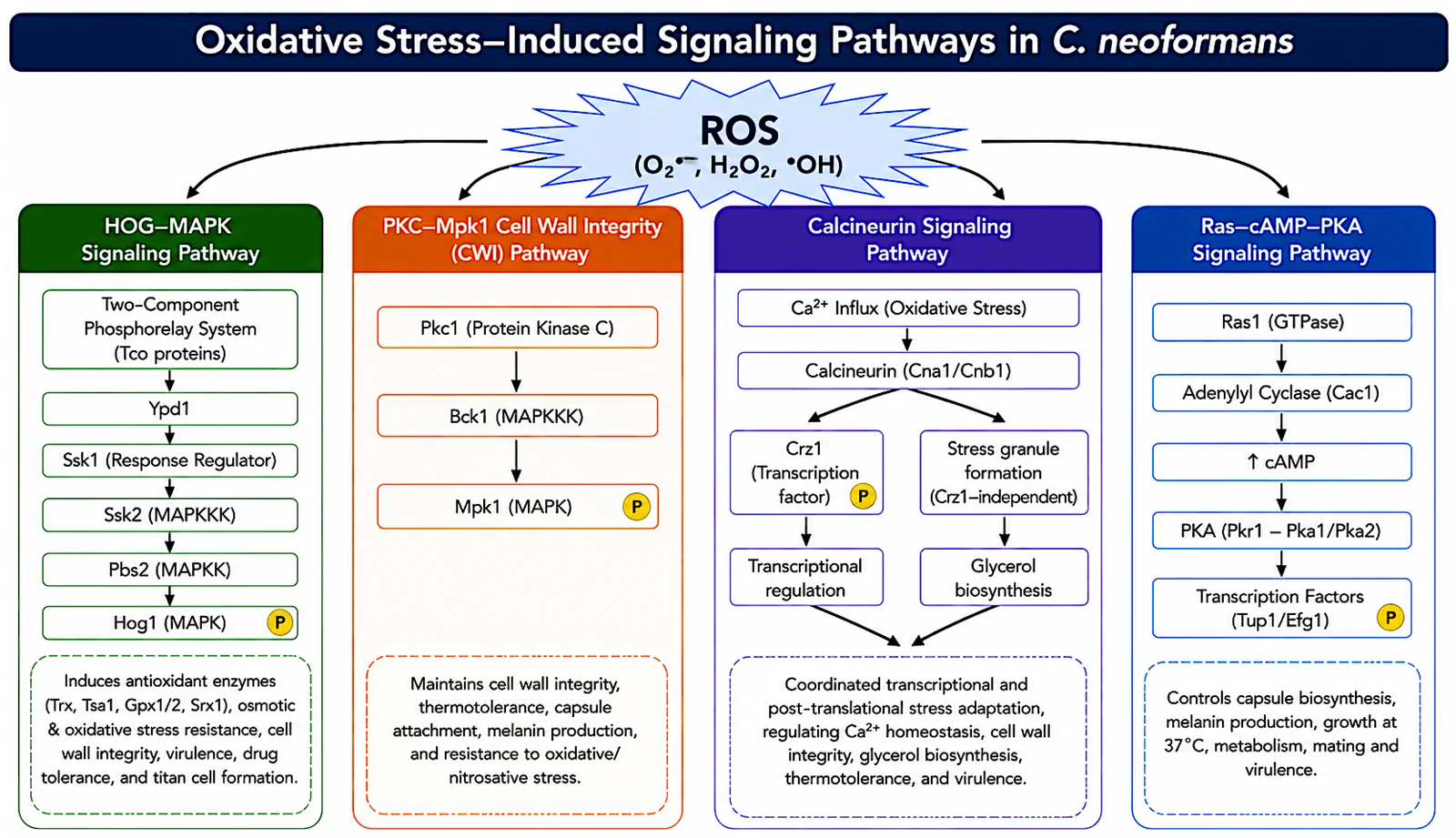
Schematic representation of the major oxidative stress-induced signaling pathways in *Cryptococcus neoformans*. Host- and mitochondria-derived ROS activate multiple stress-responsive signaling networks, including the HOG-MAPK, PKC-Mpk1 cell wall integrity, calcineurin-mediated Crz1-dependent transcriptional and stress granule-associated signaling, and Ras-cAMP-PKA pathways. Together, these pathways coordinate antioxidant defense, cell wall maintenance, glycerol biosynthesis, capsule and melanin production, titan cell formation, thermotolerance, stress adaptation, and fungal virulence.

The HOG-MAPK pathway functions as the primary oxidative stress-responsive signaling cascade, regulating antioxidant defenses and cellular adaptation to oxidative stress [78]. In parallel, the calcineurin and PKC-Mpk1 pathways cooperate to maintain calcium homeostasis, cell wall integrity, and survival under oxidative stress conditions [21]. The Ras-AMP-PKA pathway integrates environmental sensing with capsule biosynthesis, melanin production, thermotolerance, and metabolic adaptation, whereas Rim101 contributes to cell surface remodeling and immune evasion [32]. Collectively, these interconnected signaling networks coordinate oxidative stress adaptation with antioxidant defense, capsule biosynthesis, melanization, titan cell formation, virulence factor production, and host adaptation, enabling *Cryptococcus neoformans* to survive within host phagocytes, disseminate to the central nervous system, and establish persistent cryptococcal infection [54]. The major oxidative stress-responsive signaling pathways that regulate stress adaptation and virulence in *Cryptococcus neoformans* are summarized in Figure 1.

### 2.4. Candida auris

#### 2.4.1. ROS Signaling Pathways

*Candida auris* employs several conserved signaling pathways, including the cAMP/PKA, HOG MAPK, CWI MAPK, and calcineurin pathways, to regulate key virulence-associated traits such as thermotolerance, biofilm formation, morphogenesis, and multidrug resistance [22,79]. Compared with closely related *Candida* species, particularly *Candida albicans*, these pathways exhibit species-specific adaptations in *Candida auris*, with extensive signaling crosstalk contributing to stress adaptation, host persistence, and pathogenicity [80,81].

#### 2.4.2. Hog1-Dependent High-Osmolarity Glycerol (HOG) Pathway

The HOG pathway is a major ROS-responsive signaling pathway and serves as a central regulator of oxidative stress adaptation in *Candida auris* [82]. The stress-activated protein kinase (SAPK) Hog1 plays a critical role in oxidative stress tolerance, cell wall integrity, and virulence. Hog1 is activated in response to diverse oxidative stressors, including exogenous H_2_O_2_, phagocyte-derived ROS, and antifungal drug-induced redox stress [83]. Upon oxidative stress, the two-component response regulator Ssk1 transduces environmental stress signals to the MAPK kinase (MAPKK) Pbs2, which phosphorylates the conserved Thr-Gly-Tyr (TGY) motif of Hog1. Phosphorylated Hog1 subsequently translocates to the nucleus, where it interacts with stress-responsive transcription factors to regulate the expression of oxidative stress-responsive genes [84]. Activated Hog1 promotes cell wall remodeling, suppresses filamentation, and induces the expression of genes encoding adhesins (e.g., IFF4109 and ALS4), antioxidant enzymes such as catalases, and additional stress-protective proteins [85]. Through the integration of multiple oxidative stress signals into a coordinated Hog1-dependent response, the HOG pathway enhances antioxidant defense, promotes immune evasion, and supports skin colonization, persistence on abiotic surfaces, and systemic virulence in *Candida auris* [41].

#### 2.4.3. cAMP/PKA Pathway

The cAMP-protein kinase A (cAMP/PKA) pathway is a key regulator of growth, stress adaptation, biofilm formation, and virulence in *Candida auris* [46]. Signal transduction is primarily mediated by the small GTPase Ras1 and its upstream regulators, including the guanine nucleotide exchange factor Cdc25, which activates Ras1, and the GTPase-activating protein Ira2, which negatively regulates Ras1 activity [29]. In contrast, the G-protein-coupled receptor Gpr1 and the Gα subunit Gpa2 play relatively minor roles in cAMP signaling in *Candida auris* [62]. Activated Ras1 stimulates the adenylyl cyclase Cyr1, which catalyzes the conversion of ATP to cAMP. The resulting increase in intracellular cAMP promotes its binding to the regulatory subunit Bcy1, leading to the release and activation of the catalytic PKA subunits Tpk1 and Tpk2. Activated PKA phosphorylates downstream targets involved in ergosterol biosynthesis (e.g., ERG11), biofilm-associated adhesins (ALS4, SCF1, and IFF4109), hyphal growth, and secreted aspartyl proteinases (e.g., Sapa3), thereby contributing to antifungal resistance and virulence [23]. The pathway is negatively regulated by phosphodiesterases, with the high-affinity phosphodiesterase Pde2 serving as the primary regulator and the low-affinity phosphodiesterase Pde1 providing secondary control through cAMP degradation [86]. Hyperactivation of the cAMP/PKA pathway, as observed in *pde2Δ* and *bcy1Δ* mutants, results in thermosensitivity at 42–43 °C, reduced glycogen accumulation, increased susceptibility to nutrient limitation, diminished virulence in murine infection models, and reduced resistance to amphotericin B, highlighting the pathway as a potential therapeutic target through PKA inhibition or modulation of Pde2 activity [46]. Conversely, disruption of pathway components, such as *RAS1* or *CYR1*, increases tolerance to azole and echinocandin antifungals [87]. In addition, the cAMP/PKA pathway promotes biofilm development by upregulating adhesion-associated genes, including ALS4 and PGA7. Consistent with this role, *pde1Δ* and *pde2Δ* mutants exhibit enhanced biofilm formation compared with the wild-type strain [88]. Overall, the cAMP/PKA pathway integrates oxidative stress adaptation with cellular growth, metabolic regulation, biofilm development, and antifungal resistance, thereby contributing to the environmental persistence and pathogenic potential of *Candida auris* [23].

#### 2.4.4. Cell Wall Integrity (CWI)-MAPK Pathway

The CWI-MAPK pathway is a key regulator of cell wall homeostasis and contributes significantly to antifungal resistance in *Candida* [85]. In response to cell wall damage, particularly those caused by echinocandins, the pathway converts stress signals into compensatory transcriptional and structural responses that reinforce the cell wall and limit antifungal drug efficacy [81]. Cell wall damage resulting from echinocandin-mediated inhibition of β-1,3-glucan synthesis is detected by membrane-associated cell wall sensors, including Mid2- and Wsc1-like receptors [85]. Signal perception activates the small GTPase Rho1 and Pkc1, initiating a conserved MAPK signaling cascade in which the MAPKKK Bck1 phosphorylates MAPKKs (Mkk1/Mkk2-like), ultimately leading to activation of the Mkc1-related MAPK [89]. Activation of the CWI pathway induces the expression of chitin synthases and other cell wall remodeling enzymes, resulting in increased chitin deposition that compensates for β-1,3-glucan depletion and restores cell wall integrity during echinocandin exposure [90]. In addition to strengthening the cell wall, the pathway promotes glucan remodeling and contributes to biofilm matrix development, thereby enhancing tolerance to antifungal stress [89]. The CWI pathway also functionally interacts with the HOG and calcineurin signaling pathways to coordinate cell wall remodeling, prevent excessive wall rigidification, and maintain cellular homeostasis under stress conditions. This signaling crosstalk supports biofilm-associated resilience, multidrug resistance, and virulence in *Candida auris* [41]. Beyond its role in antifungal resistance, activation of the CWI pathway enables *Candida auris* to preserve cell wall integrity during oxidative stress, thereby enhancing survival against host immune defenses and oxidative damage [91].

#### 2.4.5. Calcineurin Pathway

Through a specific phosphatase-transcription-factor axis, the calcineurin pathway in *Candida auris* functions as a calcium and stress-responsive signaling module that incorporates environmental signals into cell-wall and membrane integrity, thermotolerance, and antifungal resistance [42]. The pathway is based on the heterodimeric calcium/calmodulin-dependent protein phosphatase calcineurin, which consists of a catalytic subunit Cna1 and a regulatory subunit Cnb1 [92]. High temperature, azole or echinocandin-induced membrane and cell-wall stress, and other environmental disturbances cause calcium influx, which activates this pathway [29]. Activated calcineurin dephosphorylates the transcription factor Crz1 (partially) and likely additional calcineurin-dependent regulators that control ion homeostasis, membrane and cell-wall ss-response [43]. Thus, the calcineurin-Crz1 axis is crucial for osmotic-thermal stress adaptation, antifungal resistance, and in vivo persistence in *Candida auris* because loss of Cna1 or Cnb1 (cna1Δ/cnb1Δ) eliminates extreme thermotolerance, compromises cell-membrane and cell wall integrity, causes hypersensitivity to azoles and echinocandins, and eliminates virulence in Drosophila and murine models [29]. Calcineurin signaling contributes significantly to oxidative stress adaptation by maintaining calcium homeostasis and cell integrity, thereby enhancing antifungal tolerance and persistence in *Candida auris* [29].

#### 2.4.6. Integration of Oxidative Stress Responsive Signaling Networks in *Candida auris*

**Figure 2:**
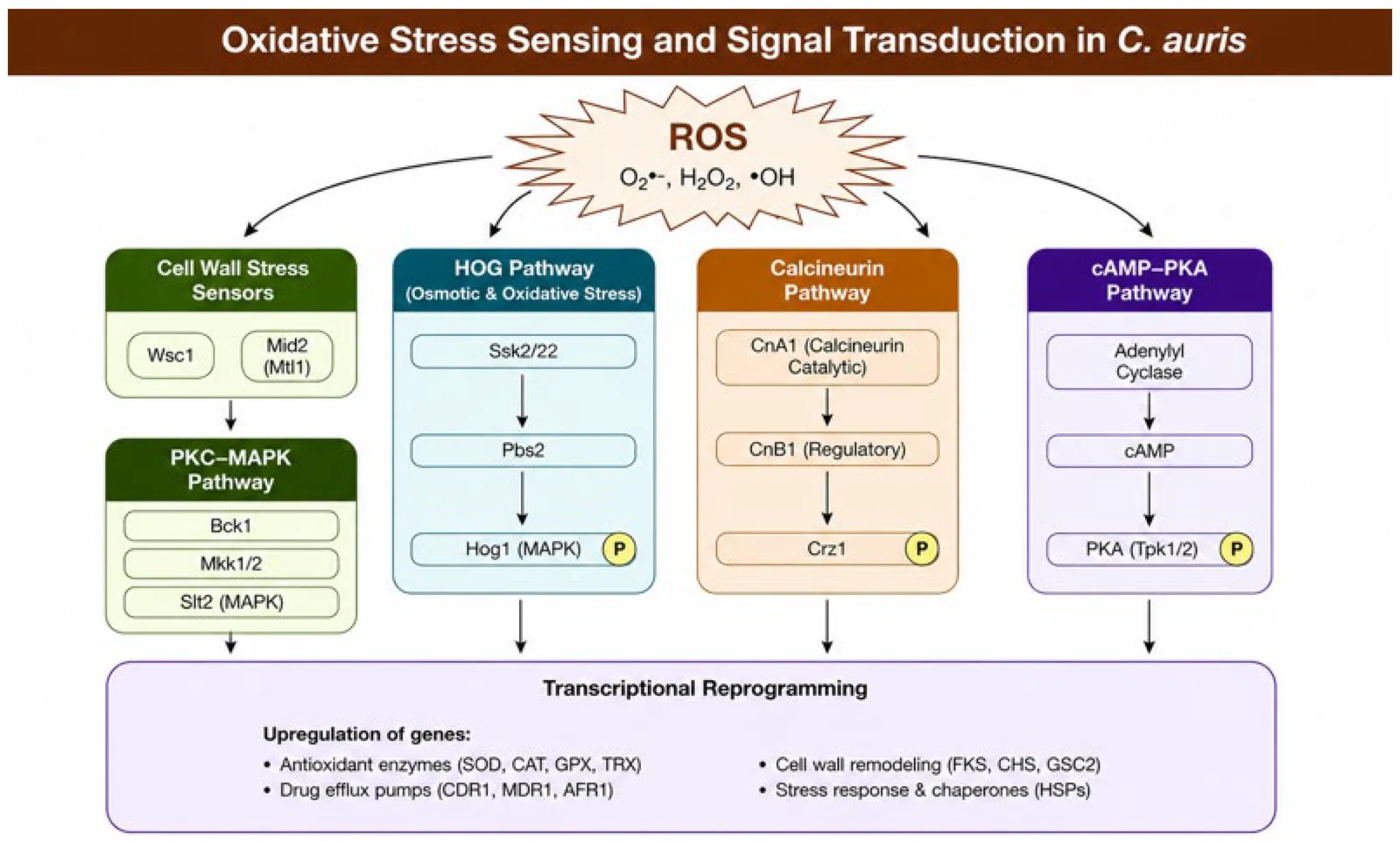
Schematic overview of oxidative stress sensing and signaling pathways in *Candida auris*. ROS activate conserved stress-response pathways, including the PKC-MAPK cell wall integrity pathway, HOG-MAPK cascade, calcineurin signaling, and the cAMP-PKA pathway. These signaling networks induce transcriptional reprogramming that upregulates antioxidant defenses, cell wall remodeling, stress-response proteins, and multidrug efflux pumps, thereby enhancing oxidative stress tolerance, thermotolerance, antifungal resistance, biofilm formation, and persistence during host infection.

Oxidative stress adaptation in *Candida auris* is mediated through the coordinated interplay of multiple signaling pathways, including the HOG-MAPK, cAMP-PKA, calcineurin, and cell wall integrity (CWI)-MAPK pathways, together with conserved antioxidant defense systems [46,83]. Among these, the HOG-MAPK pathway functions as the primary oxidative stress-responsive signaling cascade by regulating genes involved in ROS detoxification, environmental stress adaptation, and antifungal tolerance [83]. In parallel, Cap1-dependent transcriptional regulation and thioredoxin-mediated antioxidant systems maintain intracellular redox homeostasis and protect cells from oxidative damage [8]. Calcineurin and CWI-MAPK signaling cooperate to preserve cell wall integrity and promote cell survival under oxidative stress, whereas the cAMP-PKA pathway integrates oxidative stress adaptation with metabolic regulation, biofilm development, and persistence [29,93]. Compared with *Candida albicans*, oxidative stress signaling in *Candida auris* is more closely linked to multidrug resistance, environmental persistence, and enhanced tolerance to antifungal agents [79]. Collectively, the coordinated crosstalk among these signaling networks enables *Candida auris* to adapt to prolonged oxidative stress while maintaining cellular fitness, persistence, and virulence across diverse host environments [94,95]. The major oxidative stress-responsive signaling pathways and their roles in stress adaptation, antifungal resistance, and virulence in *Candida auris* are summarized in Figure 2.

### 2.5. Aspergillus fumigatus

#### 2.5.1. ROS Signaling Pathways

ROS signaling in *Aspergillus fumigatus* regulates ROS production, sensing, detoxification, and downstream responses involved in fungal development, stress adaptation, and virulence [9,96].

#### 2.5.2. RacA-Nox Pathway

The RacA-Nox pathway functions as a redox-dependent regulator of polarized growth and conidiophore development in *Aspergillus fumigatus*. In this pathway, the small GTPase RacA activates the NADPH oxidases NoxA and NoxB, generating localized ROS bursts within conidiophore vesicles [97]. These localized gradients of hydrogen peroxide (H_2_O_2_) and superoxide promote the transition of phialides from isotropic to polarized growth, thereby establishing apical dominance and ensuring directional hyphal extension rather than diffuse growth across the vesicle surface [37,98].

The RacA-Nox pathway operates largely independently of the brlA-abaA-wetA transcriptional cascade, which regulates developmental cell fate and conidial differentiation. While the brlA-abaA-wetA pathway specifies developmental identity, RacA-Nox signaling primarily controls the spatial organization required for proper conidiophore morphogenesis [99]. Loss of racA abolishes ROS production within conidiophore vesicles, resulting in a characteristic “barren” phenotype in which conidiophore stalks and swollen vesicles develop, but phialide outgrowth and conidium formation fail to occur [53]. Similarly, treatment with the NADPH oxidase inhibitor diphenyleneiodonium chloride (DPI) inhibits polarized growth and phenocopies the racA deletion phenotype by blocking phialide elongation [100]. Interestingly, carbon starvation induces compensatory ROS production that partially restores phialide emergence, suggesting the existence of alternative ROS-generating mechanisms during developmental stress [55]. Unlike antioxidant defense pathways that primarily detoxify ROS, the RacA-Nox pathway exploits controlled ROS production as a developmental signaling mechanism to regulate polarized hyphal growth, conidiation, and morphogenesis. This pathway highlights the dual role of ROS in *Aspergillus fumigatus* as both signaling molecules that coordinate fungal development and oxidative stressors that require tight cellular regulation [37].

#### 2.5.3. AfYap1-AfSkn7 Oxidative Stress Regulatory Network

The AfYap1-AfSkn7 regulatory network constitutes a major transcriptional defense system that protects *Aspergillus fumigatus* against intracellular and host-derived ROS [25,101,102,103]. AfYap1, an AP-1-like basic leucine zipper (bZIP) transcription factor, senses oxidative stress through the oxidation of conserved cysteine residues. Oxidative activation promotes its nuclear translocation, where it induces the expression of antioxidant genes encoding superoxide dismutases (SOD1-SOD4), catalases (CatA, CatB/P, and CatC), thioredoxins (TrxA and TrxB), peroxiredoxins (Prx1), and components of the glutathione system [48,101]. The coordinated induction of these antioxidant enzymes restores intracellular redox homeostasis by detoxifying superoxide and hydrogen peroxide (H_2_O_2_) and by repairing oxidatively damaged proteins [104]. Deletion of afyap1 results in intracellular ROS accumulation and increased sensitivity to H_2_O_2_ and menadione, whereas constitutive activation of AfYap1 enhances oxidative stress tolerance [101]. However, deletion of afyap1 does not produce a marked reduction in virulence in the classical murine model of invasive aspergillosis [103]. *AfSkn7*, a response regulator involved in oxidative stress signaling, functions in parallel with *AfYap1* to coordinate antioxidant defense. It directly binds to the promoters of genes encoding catalases (*CatA/P*), superoxide dismutases, and thioredoxin reductase (*TrxR*), thereby promoting a coordinated transcriptional response to oxidative stress. Loss of afskn7 impairs resistance to oxidative stress and significantly attenuates virulence [105,106]. Together, *AfYap1* and *AfSkn7* orchestrate the transcriptional activation of antioxidant defense genes, enabling *Aspergillus fumigatus* to withstand host-derived oxidative stress, maintain intracellular redox homeostasis, and promote fungal survival during infection [101,103].

#### 2.5.4. OxrA-Mediated Mitochondrial Oxidative Stress Protection Mechanism

*OxrA*, a homolog of the eukaryotic oxidation resistance 1 (*OXR1*) protein, plays a key role in maintaining mitochondrial redox homeostasis in *Aspergillus fumigatus* [49]. Unlike superoxide dismutases (SODs), which directly detoxify ROS, *OxrA* functions through a post-translational mechanism by stabilizing catalase enzymes, particularly *CatB*, against ROS-mediated inactivation [50]. Deletion of *oxrA* results in elevated intracellular ROS levels, impaired catalase activity, increased sensitivity to hydrogen peroxide (H_2_O_2_), and attenuated virulence, as evidenced by reduced pulmonary fungal burden and lower levels of lactate dehydrogenase (LDH) release and inflammatory cytokines in infected hosts [31]. Restoration of *CatB* expression rescues the *ΔoxrA* phenotype, confirming that the protective function of *OxrA* is primarily mediated through the maintenance of catalase activity [25,50]. By preserving catalase function and mitochondrial redox homeostasis during oxidative stress, *OxrA* enhances fungal fitness, oxidative stress tolerance, and pathogenicity in *Aspergillus fumigatus*.

#### 2.5.5. SakA/HOG Pathway

The SakA/HOG pathway, the functional homolog of the *Hog1* MAPK signaling cascade in yeast, serves as a central regulator of oxidative and osmotic stress adaptation in *Aspergillus fumigatus* [26,107]. Oxidative and osmotic stress signals are transmitted through upstream MAPK kinase components, including Pbs2/PbsB, leading to phosphorylation and activation of the *Hog1* ortholog SakA [53]. Activated SakA subsequently translocate to the nucleus, where it regulates the expression of genes involved in osmoadaptation, glycerol biosynthesis, antioxidant defense, and cellular stress adaptation [108]. Loss of sakA increases susceptibility to oxidative stress, demonstrating its essential role in ROS tolerance. SakA signaling also interacts with the cell wall integrity (CWI) pathway through MpkA- and Rho1-dependent signaling to coordinate cell wall remodeling during oxidative stress, thereby promoting cellular integrity under adverse environmental conditions [108]. Compared with yeast pathogens, SakA signaling in *Aspergillus fumigatus* is closely associated with filamentous growth, environmental adaptation, and invasive hyphal development. Through the integration of oxidative stress responses with morphogenesis and cell wall remodeling, the SakA/HOG pathway promotes fungal survival and persistence during host infection [107].

#### 2.5.6. Integration of Oxidative Stress Responsive Signaling Networks in *Aspergillus fumigatus*

**Figure 3:**
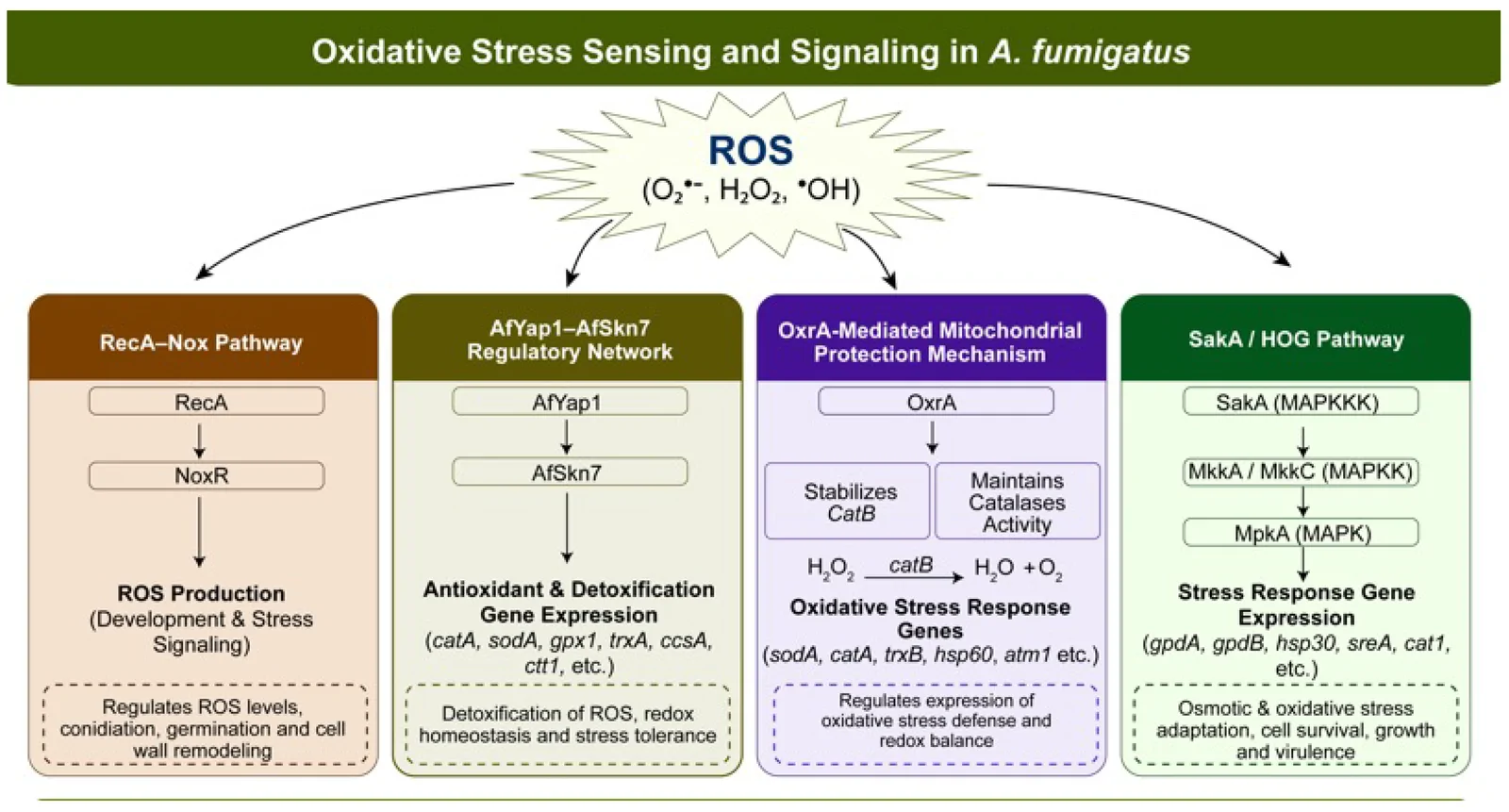
Schematic representation of oxidative stress signaling pathways in *Aspergillus fumigatus*. ROS generated during host infection activate multiple oxidative stress-responsive signaling networks, including the SakA/HOG-MAPK pathway, AfYap1- and AfSkn7-mediated transcriptional regulation, RacA-Nox signaling, the PKC-MAPK cell wall integrity pathway, calcineurin signaling, and mitochondrial OxrA-mediated protection. Coordinated activation of these pathways promotes antioxidant defense, polarized hyphal growth, cell wall remodeling, mitochondrial homeostasis, conidiation, stress adaptation, and fungal virulence under oxidative stress conditions.

In *Aspergillus fumigatus*, adaptation to oxidative stress is mediated by an integrated signaling network comprising the HOG-MAPK (SakA), cAMP-PKA, calcineurin, cell wall integrity (CWI)-MAPK, AfYap1-AfSkn7 regulatory network, RacA-Nox signaling, and the OxrA-mediated mitochondrial protection pathway [109]. Among these, the HOG-MAPK pathway serves as the primary stress-responsive signaling cascade, coordinating antioxidant gene expression and cellular adaptation to host-derived ROS [107]. In parallel, AfYap1 and AfSkn7 cooperatively regulate the expression of catalases, superoxide dismutases, thioredoxins, and other antioxidant proteins to maintain intracellular redox homeostasis [103]. Calcineurin and the CWI-MAPK pathway function together to preserve calcium homeostasis, promote cell wall remodeling, and maintain hyphal integrity during oxidative stress, whereas the cAMP-PKA pathway integrates oxidative stress adaptation with conidial germination, polarized hyphal growth, metabolism, and virulence [110]. The RacA-Nox pathway regulates localized ROS production required for polarized growth and conidiation, while the OxrA-mediated mitochondrial protection pathway preserves catalase activity, mitochondrial function, and intracellular redox balance under oxidative stress conditions [111]. Collectively, these interconnected signaling networks coordinate antioxidant defense, fungal development, stress adaptation, and virulence, thereby enabling *Aspergillus fumigatus* to survive and persist within the oxidative environment encountered during host infection [25,109,110]. The major oxidative stress-responsive signaling pathways and their roles in stress adaptation and virulence in *Aspergillus fumigatus* are illustrated in Figure 3.

### 2.6. Candida albicans

#### 2.6.1. ROS Signaling Pathways

*Candida albicans* employs conserved oxidative stress-responsive signaling pathways that regulate redox homeostasis, morphogenesis, biofilm formation, and virulence. These interconnected pathways coordinate antioxidant defense and stress adaptation, enabling the fungus to withstand host-derived oxidative stress and establish infection [10,112].

#### 2.6.2. Two-Component Signal Transduction System (TCS)

*Candida albicans* employ a two-component signal transduction system (TCS) to detect and respond to diverse environmental stimuli, including osmotic stress, oxidative stress, and the quorum-sensing molecule farnesol [113,114]. The TCS consists of three hybrid histidine kinases-Sln1p, which primarily regulates osmoregulation; Chk1p (Nik1p), which is involved in quorum sensing and cell wall integrity; and Cos1p together with the phosphotransfer protein Ypd1p and the response regulators Ssk1p, Skn7p, and Crr1p [115]. Upon sensing environmental changes, the histidine kinases undergo autophosphorylation and transfer phosphoryl groups to Ypd1p, which subsequently activates the response regulators. These regulators initiate downstream signaling pathways, including the Hog1 MAPK cascade, to coordinate adaptive stress responses [116]. Farnesol produced when the cell population exceeds approximately 10^6^ cells mL^−1^ functions as a negative regulator of hyphal morphogenesis. Chk1p serves as the primary sensor for farnesol, as *chk1Δ* mutants exhibit constitutive filamentation even at high cell densities and fail to respond to farnesol-mediated inhibition of filamentation in both planktonic cultures and biofilms. These findings provided the first evidence that a two-component signaling protein regulates quorum sensing in a eukaryotic organism [115,117]. Deletion of the non-histidine kinase domains of *CHK1* significantly reduces the infectivity of *Candida albicans* in an oral mucosal infection model, demonstrating a direct role for TCS signaling in host tissue colonization and pathogenicity [118]. In addition, the Chk1p-Ypd1p-Ssk1p phosphorelay interacts with the cAMP-PKA and MAPK signaling pathways to regulate the expression of hyphal adhesins, including HWP1 and ALS3, thereby promoting morphogenesis and biofilm development [117]. Overall, the TCS integrates environmental and oxidative stress signals with quorum sensing to coordinate adaptive transcriptional responses that support morphogenesis, biofilm formation, stress tolerance, and virulence in *Candida albicans* [10].

#### 2.6.3. Calcium-Calcineurin Signaling Pathway

The calcium-calcineurin signaling pathway is activated by an increase in intracellular Ca^2+^ levels [70]. Calcium ions bind to calmodulin (CaM), forming a Ca^2+^-CaM complex that activates calcineurin, a Ca^2+^/calmodulin-dependent serine/threonine phosphatase (PP2B) through its regulatory subunit CnB [119]. Activated calcineurin subsequently dephosphorylates the transcription factor Crz1, promoting its nuclear translocation, where it regulates the expression of genes involved in cation homeostasis (Mg^2+^, Ca^2+^, K^+^, and Mn^2+^ transport), cell wall remodeling, and stress adaptation [44]. Crz1 functions as the principal mediator of the calcineurin-dependent transcriptional response in *Candida albicans*. Genome-wide analyses have demonstrated calcineurin-dependent nuclear localization of Crz1 and identified approximately 60 genes whose expression depends on both calcineurin and Crz1. However, calcineurin-deficient mutants exhibit greater attenuation of virulence than *crz1Δ/Δ* mutants, indicating that calcineurin also regulates Crz1-independent pathways that contribute to fungal pathogenicity [44]. The calcineurin-Crz1 signaling axis promotes tolerance to echinocandins and azoles by regulating cell wall remodeling, while the broader calcineurin pathway enhances hyphal development, biofilm formation, and escape from macrophage-mediated killing during infection [120]. Overall, calcineurin signaling integrates calcium homeostasis with oxidative stress adaptation and cell wall remodeling, thereby enhancing stress tolerance, antifungal resistance, and virulence in *Candida albicans* [92,119].

#### 2.6.4. cAMP-PKA Signaling Pathway

The cyclic AMP-protein kinase A (cAMP-PKA) signaling pathway integrates diverse environmental cues, including serum, glucose, CO_2_, and temperature, through the adenylyl cyclase Cyr1, whose activity is stimulated by the small GTPase Ras1. Activated Cyr1 catalyzes the conversion of ATP to cAMP (cAMP), thereby initiating downstream signaling events [121]. Elevated intracellular cAMP binds to the regulatory subunit Bcy1, triggering the release and activation of the catalytic PKA subunits Tpk1 and Tpk2. These kinases phosphorylate downstream targets, including the transcription factor Efg1, which promotes hyphal morphogenesis and regulates the expression of virulence-associated genes [122,123]. Although Tpk1 and Tpk2 have partially redundant functions, they also perform distinct roles during morphogenesis. Tpk1 is primarily required for hyphal development on solid media, whereas Tpk2 regulates hyphal formation in liquid media and promotes invasive growth. Simultaneous deletion of *TPK1* and *TPK2* completely abolishes filamentation under in vitro conditions [121]. Pathway activity is negatively regulated by the phosphodiesterases Pde1 (low-affinity) and Pde2 (high-affinity), which degrade intracellular cAMP. Deletion of *PDE2* results in hyperfilamentation but reduced virulence due to impaired adhesion, whereas *pde1Δ/Δ* mutants retain normal filamentation [121]. Intravital imaging studies have demonstrated that strains lacking Cyr1 or both PKA catalytic subunits (Tpk1 and Tpk2) retain the ability to undergo filamentation during in vivo infection despite being completely defective in filamentation under in vitro conditions. These findings indicate that cAMP-independent signaling pathways play a predominant role in regulating filamentation within the host environment, while the downstream transcription factors Efg1 and Nrg1 continue to function as key positive and negative regulators of filamentation, respectively [45]. Overall, the cAMP-PKA pathway integrates oxidative stress adaptation with morphogenesis, biofilm development, and environmental sensing, enabling *Candida albicans* to rapidly adapt to changing host conditions and maintain pathogenicity.

#### 2.6.5. HOG-MAPK Signaling Pathway

The high osmolarity glycerol (HOG)-MAPK pathway is the principal signaling cascade that mediates adaptation to osmotic and oxidative stress in *Candida albicans*. Environmental stress signals are transmitted through the MAPKKK Ssk2 to the MAPK kinase Pbs2, which subsequently phosphorylates and activates the stress-activated MAPK Hog1 [116]. The interaction between Ssk1 and Ssk2 facilitates Ssk2-Pbs2 signaling and contributes to the maintenance of basal Hog1 phosphorylation. Under oxidative stress, however, Hog1 activation can occur independently of the two-component signaling system. Hydrogen peroxide (H_2_O_2_) promotes the oxidation and inactivation of protein tyrosine phosphatases that normally suppress Hog1 activity, a process that is partially regulated by the thioredoxin system [124]. Following phosphorylation, Hog1 translocate to the nucleus, where it regulates the expression of stress-responsive genes and coordinates cell cycle progression in response to oxidative stress [125]. Using a quadruple MAPK deletion mutant, the Hog1 is the dominant kinase for both osmotic and oxidative stress adaptation, establishing it as an indispensable virulence determinant [125]. Overall, Hog1 integrates oxidative stress adaptation with morphogenesis, stress-responsive gene expression, and immune evasion, thereby promoting the survival and pathogenicity of *Candida albicans* during host infection.

#### 2.6.6. Integration of Oxidative Stress Responsive Signaling Networks in *Candida albicans*

**Figure 4:**
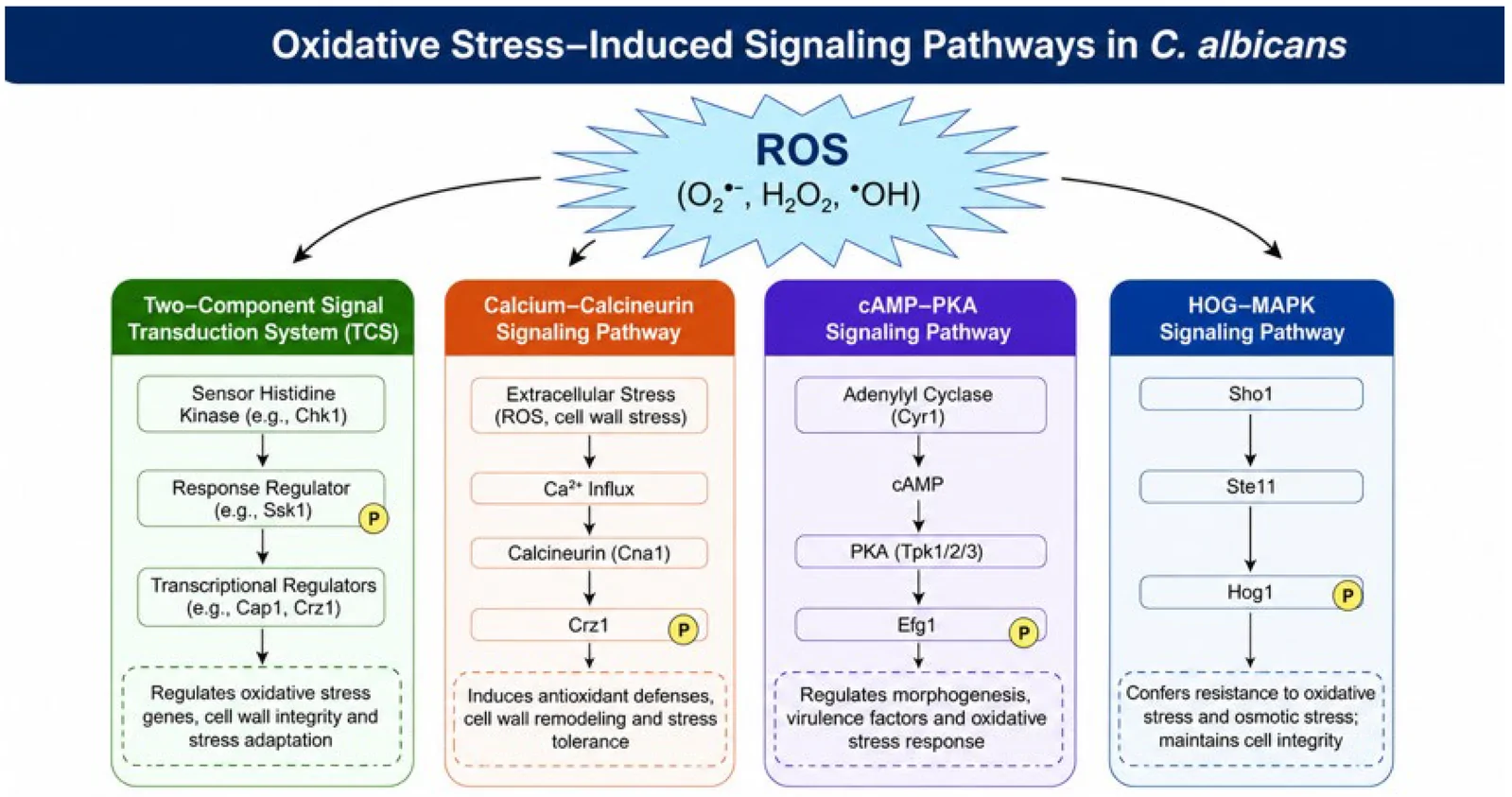
Schematic representation of oxidative stress-induced signaling pathways in *Candida albicans*. ROS activate multiple interconnected signaling pathways, including the two-component signaling system (TCS), HOG-MAPK, calcium-calcineurin, and cAMP-PKA pathways. These pathways regulate transcriptional responses involved in antioxidant defense, morphogenesis, cell wall remodeling, biofilm formation, stress adaptation, and virulence, enabling *Candida albicans* to survive oxidative stress encountered during host infection.

In *Candida albicans*, adaptation to oxidative stress is orchestrated through extensive crosstalk among the HOG-MAPK, Cap1-mediated oxidative stress response, cAMP-PKA, calcineurin, and cell wall integrity (CWI)-MAPK pathways [126]. Among these, the HOG-MAPK pathway serves as the primary oxidative stress-responsive signaling cascade, coordinating the expression of antioxidant enzymes and other stress-responsive genes [124]. In parallel, the Cap1-Gpx3-Ybp1 regulatory module functions as the principal ROS-sensing mechanism by coupling intracellular redox changes to the transcriptional activation of antioxidant defense genes. The thioredoxin system further contributes to redox homeostasis by regulating Cap1 activity and maintaining the intracellular redox balance [36]. Calcineurin and the CWI-MAPK pathway cooperate to preserve calcium homeostasis, maintain cell wall integrity, and promote cell survival under oxidative stress [44,126]. Concurrently, the cAMP-PKA pathway integrates oxidative stress adaptation with morphogenetic switching, biofilm development, and metabolic reprogramming, enabling fungal cells to balance stress resistance with growth and virulence [10,43]. Collectively, these interconnected signaling networks coordinate antioxidant defense, DNA repair, morphogenesis, immune evasion, and stress adaptation, thereby promoting fungal survival, persistence, and pathogenicity during host infection cAMP-PKA [45,96,112]. A schematic overview of the oxidative stress-responsive signaling pathways that coordinate stress adaptation and virulence in *Candida albicans* is presented in Figure 4.

A comparative summary of the major oxidative stress signaling pathways, their key regulatory components, and their primary biological functions across *Cryptococcus neoformans*, *Candida auris*, *Aspergillus fumigatus*, and *Candida albicans* is presented in Table 3.

### 2.7. Comparative Analysis of Oxidative Stress Signaling in Cryptococcus neoformans, Candida auris, Aspergillus fumigatus, and Candida albicans

Although oxidative stress response mechanisms are broadly conserved among fungal pathogens, significant differences exist in how individual species sense, respond to, and exploit ROS during host infection [10]. All four pathogens possess antioxidant defense systems, including catalases, superoxide dismutases, thioredoxin systems, and glutathione-dependent pathways, which collectively maintain intracellular redox homeostasis under oxidative stress conditions [70]. Conserved signaling pathways such as HOG-MAPK, calcineurin, and cAMP-PKA further coordinate adaptation to oxidative stress, although their downstream regulatory networks and biological functions differ considerably among species [77,127,128].

Despite these similarities, each pathogen exhibits distinct oxidative stress adaptations [129]. *Cryptococcus neoformans* possesses a unique oxidative stress adaptation strategy centered on its polysaccharide capsule and melanin production, both of which provide effective protection against host-derived ROS. Oxidative stress responses are coordinated through the HOG-MAPK pathway together with the two-component signaling (TCS) system, calcineurin, cAMP-PKA, and PKC-Mpk1 cell wall integrity signaling [33,130]. These pathways regulate antioxidant defenses, thermotolerance, intracellular survival within macrophages, and central nervous system dissemination, making oxidative stress adaptation an essential determinant of cryptococcal virulence [66]. In *Candida auris*, oxidative stress signaling is closely associated with multidrug resistance, thermotolerance, environmental persistence, and biofilm formation [41,94]. The Hog1 pathway plays a particularly important role in coordinating stress adaptation and survival under antifungal pressure [84]. *Aspergillus fumigatus* possesses specialized oxidative stress mechanisms associated with filamentous growth and development, including the RacA-Nox pathway that regulates polarized growth and conidiation through localized ROS generation [53]. In contrast, *Candida albicans* integrates oxidative stress signaling with morphogenetic switching, macrophage escape, and biofilm development through coordinated regulation by the Hog1, Cap1, and cAMP-PKA signaling networks [30,125,131].

The relationship between oxidative stress adaptation and antifungal resistance also differs among these pathogens. In *Cryptococcus neoformans*, oxidative stress resistance is primarily mediated by capsule and melanin, which protect fungal cells from phagocyte-derived ROS and facilitate intracellular persistence. For *Candida auris*, oxidative stress signaling contributes directly to multidrug tolerance and persistence, whereas in *Aspergillus fumigatus*, oxidative stress responses primarily support survival under host-imposed environmental stresses. In *Candida albicans*, oxidative stress signaling is intimately linked to morphogenesis and virulence-associated traits that promote host colonization and invasion [14,22,50,89,115]. Collectively, these observations suggest that while core oxidative stress signaling pathways are conserved, pathogen-specific regulatory adaptations determine the outcome of host–pathogen interactions [27]. Understanding these similarities and differences may facilitate the development of antifungal therapies targeting conserved stress-response networks while simultaneously exploiting species-specific vulnerabilities. A comparative overview of oxidative stress-responsive signaling pathways, antioxidant defenses, and redox regulatory mechanisms in the four major human fungal pathogens is presented in Table 4.

## 3. Conclusions and Further Perspective

The interplay between host-derived ROS and fungal antioxidant defense systems is a major determinant of the survival and pathogenicity of the WHO priority fungal pathogens. During host–pathogen interactions, ROS performs a dual function. On the one hand, ROS acts as a potent antimicrobial defense mechanism generated by host immune cells to eliminate invading fungi. On the other hand, fungal pathogens exploit ROS as intracellular signaling molecules that regulate stress adaptation, redox homeostasis, morphogenesis, and virulence. *Cryptococcus neoformans*, *Candida auris*, *Aspergillus fumigatus*, and *Candida albicans* have each evolved sophisticated antioxidant defense systems and oxidative stress-responsive signaling networks that enable survival within the highly oxidative environment encountered during infection. Comparative analysis of these fungal pathogens demonstrates that oxidative stress signaling extends beyond a protective response and functions as a central regulator of fungal fitness, host adaptation, and pathogenicity. Although conserved signaling pathways, including HOG-MAPK, calcineurin, and cAMP-PKA, coordinate oxidative stress adaptation across these pathogens, each species has evolved distinct regulatory mechanisms that reflect its unique biology. *Cryptococcus neoformans* relies on capsule formation, melanization, and intracellular survival within macrophages; *Candida auris* couples oxidative stress adaptation with multidrug resistance, thermotolerance, and environmental persistence; *Aspergillus fumigatus* employs ROS-dependent polarized growth and mitochondrial protection mechanisms; whereas *Candida albicans* integrates oxidative stress signaling with morphogenesis, biofilm development, and immune evasion.

Importantly, several components of fungal redox homeostasis differ structurally and functionally from their mammalian counterparts, providing opportunities for selective therapeutic intervention. Conserved signaling regulators, including Hog1/SakA, calcineurin-associated pathways, the cAMP-PKA pathway, fungal-specific superoxide dismutases, and thioredoxin-dependent antioxidant systems, represent promising targets for future antifungal drug development. In addition, species-specific regulators, such as the Cap1-Gpx3-Ybp1 regulatory module in *Candida albicans*, the OxrA-mediated mitochondrial protection mechanism, and RacA-Nox pathway in *Aspergillus fumigatus*, and capsule- and melanin-associated oxidative stress responses in *Cryptococcus neoformans*, may provide opportunities for precision antifungal therapies.

Emerging evidence further suggests that fungal metabolic pathways are closely integrated with oxidative stress signaling and may represent additional therapeutic opportunities. For example, metabolic adaptation through methionine utilization has been shown to induce mild intracellular ROS accumulation, activating the Gcn2-eIF2α-Gcn4 signaling axis and thereby linking oxidative stress responses with translational regulation and cellular metabolism. These findings further highlight the close interplay between fungal metabolism and redox homeostasis, suggesting that metabolic regulators may represent promising targets for future antifungal intervention.

Despite substantial advances, several important questions remain unresolved. The quantitative thresholds that distinguish ROS-mediated signaling from oxidative damage remain poorly understood, particularly in emerging multidrug-resistant pathogens such as *Candida auris*. Furthermore, the molecular crosstalk among oxidative stress signaling, antifungal resistance, metabolic reprogramming, mitochondrial function, and cell wall remodeling requires further investigation across diverse fungal pathogens. Future studies integrating transcriptomics, proteomics, metabolomics, functional genomics, and real-time redox imaging are expected to provide a more comprehensive understanding of species-specific regulatory networks and to uncover novel therapeutic vulnerabilities. From a translational perspective, continued investigation of oxidative stress-responsive signaling pathways is expected to facilitate the identification of novel antifungal targets and support the development of innovative redox-based therapeutic strategies. Although no approved antifungal agent currently targets fungal redox homeostasis as its primary mechanism of action, several clinically used antifungal drugs, including amphotericin B and azoles, induce intracellular ROS accumulation as part of their antifungal activity. Experimental targeting of thioredoxin systems, glutathione metabolism, oxidative stress-responsive signaling pathways, and fungal antioxidant enzymes has demonstrated encouraging antifungal efficacy in preclinical studies, highlighting the therapeutic potential of redox-based strategies for overcoming emerging antifungal resistance.

In summary, the comparative analysis of *Cryptococcus neoformans*, *Candida auris*, *Aspergillus fumigatus*, and *Candida albicans* demonstrates that oxidative stress adaptation is governed by a combination of evolutionarily conserved signaling pathways and species-specific regulatory mechanisms. A deeper understanding of these shared and divergent oxidative stress-responsive networks will not only advance our knowledge of fungal pathogenesis but also accelerate the development of both broad-spectrum and species-specific antifungal therapies to address the growing global burden of invasive fungal diseases.

## Figures and Tables

**Table 1:** Classification of oxidative stress signaling pathways in fungal pathogens.

Classification	Pathway/ Regulator	Primary Physiological Function	Mechanism of Activation Under Oxidative Stress	Representative Species
Direct ROS- sensing mechanism	Cap1/Yap1-Gpx3-c	Antioxidant gene regulation and redox homeostasis	Direct oxidation of Gpx3 induces Cap1/Yap1 activation and nuclear localization	*Candida albicans*, *Candida auris*
	Skn7	Coordination of oxidative stress defense genes	Activated through redox-dependent signaling and cooperates with Cap1/Yap1	*Candida albicans*, *Aspergillus fumigatus*
	Thioredoxin (Trx1)-dependent signaling	Maintenance of cellular redox balance and regulation of stress-responsive proteins	Changes in thiol-disulfide status modulate downstream signaling components	*Candida albicans*, *Candida auris*
Indirect ROS- responsive pathway	HOG-MAPK (Hog1/SakA)	Adaptation to oxidative and environmental stress	Activated by oxidative stress-induced signaling through upstream sensor systems	*Cryptococcus neoformans*, *Candida albicans*, *Candida auris*, *Aspergillus fumigatus*
	cAMP-PKA pathway	Morphogenesis, metabolism, stress adaptation, and virulence	Activated through oxidative stress-mediated metabolic and environmental cues	*Cryptococcus neoformans*, *Candida albicans*, *Candida auris*, *Aspergillus fumigatus*
	Calcineurin pathway	Calcium homeostasis, stress adaptation, cell survival	Activated by oxidative stress-associated intracellular calcium signaling	*Cryptococcus neoformans*, *Candida albicans*, *Candida auris*, *Aspergillus fumigatus*
	Cell wall integrity (CWI)-MAPK pathway	Cell wall remodeling and maintenance	Triggered by oxidative stress-induced cell wall damage	*Cryptococcus neoformans*, *Candida albicans*, *Aspergillus fumigatus*
	UPR (unfolded protein response)	Endoplasmic reticulum proteostasis and stress adaptation	Activated by accumulation of oxidatively damaged or misfolded proteins	*Candida albicans*, *Aspergillus fumigatus*
	Mitochondrial stress response (including OxrA-mediated regulation)	Mitochondrial protection and energy homeostasis	Activated by mitochondrial ROS accumulation and oxidative damage	*Aspergillus fumigatus*

**Table 2:** Classification of oxidative stress signaling pathways in major human fungal pathogens. Oxidative stress-associated signaling pathways are grouped into conserved and species-specific mechanisms based on their mode of activation, evolutionary conservation, and contribution to oxidative stress adaptation.

Classification	Pathway/ Regulator	Mechanism of Activation Under Oxidative Stress	Species-Specific Adaptations
Conserved	HOG-MAPK (Hog1/SakA)	Activated through oxidative stress-induced signaling via upstream sensor systems and MAPK phosphorylation cascades.	Conserved in *Candida auris*, *Candida albicans*, *Aspergillus fumigatus*, and *Cryptococcus neoformans*. Associated with multidrug tolerance in *Candida auris*, morphogenesis in *Candida albicans*, stress adaptation in *Aspergillus fumigatus*, and oxidative/osmotic stress resistance and virulence in *Cryptococcus neoformans* [26,33,40,41].
	Calcineurin pathway	Activated by oxidative stress-associated intracellular Ca^2+^ signaling.	Conserved across all four pathogens. Regulates stress adaptation, cell survival, thermotolerance, and antifungal tolerance, although downstream targets differ among species [29,42,43,44].
	cAMP-PKA pathway	Activated through oxidative stress-mediated metabolic and environmental cues.	Present in all four pathogens. Regulates morphogenesis and biofilm formation in *Candida*, hyphal development in *Aspergillus fumigatus*, and capsule formation, melanin production, and virulence in *Cryptococcus neoformans* [45,46,47].
	Cap1/Yap1-Gpx3-Ybp1 oxidative stress regulatory network	Direct oxidation of Gpx3 induces Cap1/Yap1 activation and nuclear localization.	Central ROS-sensing mechanism in *Candida* species and functionally conserved through Yap1 homologs in *Aspergillus fumigatus*. *Cryptococcus neoformans* possesses AP-1-like oxidative stress regulators but lacks the canonical Cap1-Gpx3-Ybp1 module [36,48].
	Cell wall integrity (CWI)-MAPK pathway	Triggered by oxidative stress-induced cell wall damage.	Conserved in all four pathogens. Particularly important for cell wall remodeling, stress adaptation, and virulence, although pathway architecture varies among species [49,50,51,52].
Species-Specific	OxrA-mediated mitochondrial oxidative stress protection mechanism	Activated by mitochondrial ROS accumulation and oxidative damage.	Predominantly characterized in *Aspergillus fumigatus*, where OxrA stabilizes catalase activity, preserves mitochondrial integrity, and contributes to oxidative stress tolerance and virulence [50].
	RacA-Nox signaling	Activated through localized ROS generation during fungal development.	Primarily reported in *Aspergillus fumigatus*, where it regulates polarized hyphal growth, conidiation, cell wall remodeling, and developmental ROS signaling [53].

**Table 3:** Major oxidative stress signaling pathways and their biological functions in *Cryptococcus neoformans*, *Candida auris*, *Aspergillus fumigatus*, and *Candida albicans*.

Organism	Pathway	Key Components/Regulators	Primary Biological Outcome
*Cryptococcus neoformans*	HOG-MAPK pathway	Tco proteins, Ssk1, Ssk2, Pbs2, Hog1	Oxidative and osmotic stress tolerance, antioxidant gene expression, intracellular survival, capsule regulation and virulence [33].
	Calcineurin pathway	Ca^2+^, Calmodulin, Calcineurin (Cna1/Cnb1), Crz1	Calcium homeostasis, thermotolerance, oxidative stress adaptation, macrophage survival, cell wall integrity and virulence [69,71,75].
	cAMP-PKA pathway	Gpr4, Gpa1, Cac1 (adenylyl cyclase), cAMP, Pka1/Pkr1	Capsule biosynthesis, melanin production, thermotolerance, nutrient sensing and virulence [59,76].
	PKC-Mpk1 cell wall integrity (CWI) pathway	Pkc1, Bck1, Mkk2, Mpk1	Cell wall remodeling, thermotolerance, oxidative stress resistance, capsule attachment and maintenance of cell integrity during host infection [65,66,67].
*Candida auris*	HOG-MAPK pathway	Hog1 (MAPK), upstream MAPKK/MAPKKK	Environmental and oxidative stress adaptation; thermotolerance; regulation of adhesins (e.g., IFF4109); survival under hostile conditions [14,114].
	cAMP-PKA pathway	Cyr1 (adenylyl cyclase), Ras1, cAMP, PKA (Tpk1, Tpk2), Bcy1, PDE1/PDE2	Thermotolerance, growth regulation, biofilm formation, morphogenesis, stress response, antifungal resistance, adhesion, melanization [46].
	Cell wall integrity (CWI)-MAPK pathway	Pkc1-Bck1-MAPKK-Mkc1	Cell wall remodeling, β-glucan and chitin synthesis, stress adaptation and antifungal drug resistance [83,85].
	Calcineurin pathway	Calcineurin (Cna1/Cnb1), Ca^2+^ signaling components, Crz1	Calcium homeostasis, stress adaptation, drug tolerance, extreme thermotolerance (>43 °C), survival under antifungal stress [92].
*A* *spergillus fumigatus*	RacA-Nox pathway	RacA (small GTPase), NoxA/NoxB (NADPH oxidases)	Localized ROS generation, polarized hyphal growth, phialide development and conidiation [53,97,99].
	AfYap1-AfSkn7 pathway	AfYap1, AfSkn7 (transcription factors)	Oxidative stress sensing, antioxidant gene regulation and ROS detoxification [25,101,103].
	OxrA-mediated mitochondrial oxidative stress protection pathway	OxrA (mitochondrial protein)	Catalase stabilization, mitochondrial protection and maintenance of redox homeostasis by decreasing intracellular ROS [25,49,50].
	SakA/HOG-MAPK pathway	SakA (Hog1 ortholog), PbsB, SskB	Oxidative and hyperosmotic stress tolerance, cell wall remodeling and stress adaptation [53,107].
*Candida albicans*	Two-Component Signaling (TCS) pathway	Sln1p, Chk1p/Nik1p, Cos1p, Ypd1p, Ssk1p, Skn7p, Crr1p	Environmental sensing, regulation of morphogenesis, quorum sensing, biofilm formation and oxidative stress adaptation [115,118].
	Quorum sensing (Farnesol signaling)	Farnesol, Chk1p, Ypd1p, Ssk1p; downstream targets (HWP1, ALS3)	Inhibition of hyphal formation, maintenance of yeast morphology, regulation of biofilm maturation and dispersal [10,113,114].
	Calcium-calcineurin pathway	Ca^2+^, calmodulin (CaM), calcineurin (CnA/CnB), Crz1	Stress adaptation, calcium homeostasis, cell wall remodeling, antifungal tolerance and virulence [44,70].
	cAMP-PKA pathway	Cyr1 (adenylyl cyclase), Ras1, cAMP, Bcy1, Tpk1, Tpk2, Pde1, Pde2	Regulation of yeast-to-hypha transition, filamentation, environmental adaptation and virulence [45,121].
	(HOG)-MAPK pathway	MAPK, Ssk1, Ssk2	Oxidative stress adaptation with morphogenesis, stress-responsive gene expression, and immune evasion [40,124,125].

**Table 4:** Comparative overview of oxidative stress adaptation and redox regulatory networks in *Cryptococcus neoformans*, *Candida auris*, *Aspergillus fumigatus*, and *Candida albicans*. The table summarizes the major sources of ROS, principal oxidative stress signaling pathways, antioxidant defense systems, characteristic oxidative stress adaptations, contributions to virulence and antifungal resistance, and potential therapeutic targets in four clinically important fungal pathogens. The comparison highlights both conserved redox regulatory mechanisms and species-specific adaptations that facilitate survival under oxidative stress and contribute to fungal pathogenicity.

Feature	*Cryptococcus neoformans*	*Candida auris*	*Aspergillus fumigatus*	*Candida albicans*
Morphology	Encapsulated yeast	Yeast	Filamentous mold	Dimorphic yeast
Major ROS sources	Mitochondrial ETC, host oxidative burst, macrophage-derived ROS	Mitochondrial ETC, antifungal stress	Mitochondrial ETC, NADPH oxidases, environmental stress	Mitochondrial ETC, host oxidative burst
Key oxidative stress regulators	Hog1, TCS (Tco1/Tco2-Ssk1), calcineurin, cAMP-PKA, PKC-Mpk1	Hog1, Calcineurin, cAMP-PKA	SakA/HOG, AfYap1, AfSkn7, OxrA, RacA-Nox	Hog1, Cap1, calcineurin, cAMP-PKA
Major antioxidant systems	Catalases, SODs, thioredoxin, glutathione, peroxiredoxins, melanin	Catalases, SODs, thioredoxin pathways	Catalases, SODs, thioredoxin pathways	Catalases, SODs, glutathione and thioredoxin systems
Unique adaptation	Polysaccharide capsule, melanin production, intracellular survival within macrophages	Multidrug resistance and environmental persistence	ROS-regulated polarized growth and conidiation	Morphogenesis and macrophage escape
Association with virulence	Capsule formation, melanin production, macrophage survival, CNS dissemination	Biofilm formation, thermotolerance, immune evasion	Hyphal development, invasive growth	Hyphal transition, biofilm formation
Association with antifungal resistance	Melanin- and capsule-mediated stress protection, enhanced survival under host-induced oxidative stress	Strongly linked to multidrug resistance	Supports stress tolerance and survival	Contributes to drug tolerance and adaptation
Potential therapeutic targets	Hog1, calcineurin, PKC-Mpk1 pathway, capsule and melanin biosynthesis	Hog1, calcineurin, CWI signaling	SakA, OxrA, AfYap1	Cap1, Sod5, Thioredoxin system
References	[132,133]	[46,83,134,135]	[50,53,101]	[10,45,124,136]

## Data Availability

No new data were created or analyzed in this study. Data sharing is not applicable to this article.

## Abbreviations

The following abbreviations are used in this manuscript

AfYap1	*Aspergillus fumigatus* Yeast Activator Protein 1
ALS3	Agglutinin-Like Sequence 3
ALS3,4	Agglutinin-Like Sequence 3,4
ATP	Adenosine Triphosphate
Bck1	Bypass of C Kinase 1
Bcy1	Regulatory Subunit of Protein Kinase A
BER	Base Excision Repair
brlA	Bristle A
CaM	Calmodulin
cAMP-PKA	Cyclic Adenosine Monophosphate-pathway
Cap1	*Candida* AP-1-Like Transcription Factor 1
Cat1	Catalase 1
CatA/P	Catalase A/Peroxisomal Catalase
Cdc25	Cell Division Cycle Protein 25
Chk1p	Histidine Kinase Checkpoint kinase 1 protein
Chk1p/Nik1p	Histidine Kinase 1/Negative Histidine Kinase 1
Chk2	Checkpoint Kinase 2
Cna1	Calcineurin Catalytic Subunit A1
CnB	Calcineurin Regulatory Subunit B
Cnb1	Calcineurin Regulatory Subunit B1
Crr1p	*Candida* Response Regulator 1
Crz1	Calcineurin-Responsive Zinc Finger Transcription Factor 1
CWI-MAPK	Cell Wall Integrity-Mitogen-Activated Protein Kinase
cycA	Cytochrome C A
Cyr1	Cyclase-Related protein 1 (adenylyl cyclase)
Dal81	DNA-Binding Activator of Allantoin Metabolism 81
DCF	Dichlorofluorescein
DCFH-DA	2′,7′-Dichlorodihydrofluorescein Diacetate
Dpb4	DNA polymerase epsilon subunit B4
DPI	Diphenyleneiodonium
Efg1	Enhanced Filamentous Growth Protein 1
ERG11	Ergosterol Biosynthesis Gene 11
ETC	Electron Transport Chain
Glr1	Glutathione Reductase 1
GN2	Group 2 fungal pathogens
GPCR	G-Protein-Coupled Receptor
GPI	Glycosylphosphatidylinositol
Gpr1	G-Protein Receptor 1
Gpx1/2/3	Glutathione Peroxidase 1/2/3
H2DCFDA	2′,7′-Dichlorodihydrofluorescein Diacetate
H_2_O_2_	Hydrogen Peroxide
Hap43	Heme Activator Protein 43
HOCl	Hypochlorous Acid
HOG MAPK	High Osmolarity Glycerol–Mitogen-Activated Protein Kinase
Hog1	High Osmolarity Glycerol Response Kinase 1
HR	Homologous Recombination
HWP1	Hyphal Wall Protein 1
IFD	Invasive Fungal Disease
K^+^	Potassium Ion
LDH	Lactate Dehydrogenase
MAPK	Mitogen-Activated Protein Kinase
MchB	Monocarboxylate Transporter B
Mkc1	MAP Kinase of *Candida* 1
Mn^2+^	Manganese Ion
MPH1	Mutator Phenotype Helicase 1
NADPH	Nicotinamide Adenine Dinucleotide Phosphate
Nce103	Carbonic Anhydrase Nce103
NER	Nucleotide Excision Repair
NOX2	NADPH Oxidase 2
NoxA/B	NADPH Oxidase A/B
Nrg1	Negative Regulator of Growth 1
O_2_^−^	Superoxide Anion
OxrA	Oxidation Resistance Protein A
Pbs2	Polybrene sensitivity protein 2 (MAPKK)
Pbs2/PbsB	Polymyxin B Sensitivity 2/Phospho-Binding Subunit B
Pde1	Low-affinity Phosphodiesterase 1
Pde1/Pde2	Phosphodiesterase 1/2
PGA7	Putative GPI-Anchored Protein 7
Pkc1	Protein Kinase C 1
PP2B	Protein Phosphatase 2B
RacA	Ras-related C3 botulinum toxin substrate A
RAD17	Radiation Sensitive 17
RAD52	Radiation sensitive protein 52
RAD6	Radiation sensitive protein 6
Ras1	Rat Sarcoma-like GTPase 1
RDH54	RAD Homolog 54
Rho1	Ras Homolog 1
ROS	Reactive Oxygen Species
RT-qPCR	Reverse Transcription Quantitative Polymerase Chain Reaction
RTT109	Regulator of Ty1 Transposition 109
SakA	Stress-Activated Kinase A
SAPK	Stress-Activated Protein Kinase
Skn7	Suppressor of Kinase Mutant 7
Sln1p	Synthetic Lethal of N-end Rule 1 Protein
SOD	Superoxide Dismutase
SrbA	Sterol Regulatory Element-Binding Protein A
Ssk1p	Suppressor of Sensor Kinase 1 Protein
Ssk2	Suppressor of Sensor Kinase 2
Stp2	Suppressor of tryptophan permease 2
TBARS	Thiobarbituric Acid Reactive Substances
TGY	Threonine Glycine Tyrosine motif
TLR2	Toll-Like Receptor 2
TLR4	Toll-Like Receptor 4
Tpk2	Catalytic Subunit of 2
Trr1	Thioredoxin Reductase 1
Tsa1	Thiol-specific Antioxidant 1 (Peroxiredoxin)
wetA	Wet-white A
WHO	World Health Organization
Wsc1	Wall Stress Component 1
Ybp1	Yap1-Binding Protein 1
